# Molecular research: the effect of black fig (*Ficus carica* L.) leaf extract on inflammation in punch skin biopsy

**DOI:** 10.1007/s10787-025-01798-8

**Published:** 2025-05-31

**Authors:** Sinem Gültekin Tosun, Esra Balcıoğlu, Korhan Arslan, Gülce Yıldız, Bilal Akyüz

**Affiliations:** 1 Department of Veterinary Genetics and Biotechnology, Institute of Health Science, Erciyes University, Kayseri, Türkiye; 2 Department of Histology and Embryology, Faculty of Medicine, Erciyes University, Kayseri, Türkiye; 3 Department of Genetics, Faculty of Veterinary Medicine, Erciyes University, Kayseri, Türkiye

**Keywords:** Fig, IL-1β, IL-6, Skin, TNF-α, Wound healing

## Abstract

Although the pharmacological benefits of fig (*Ficus carica*) leaf, belonging to the Moraceae family, such as antioxidant, anti-inflammatory, antibacterial, antifungal, antidiabetic, and anticancer properties are well-documented, studies on their potential effects on wound healing and the molecular mechanisms involved in this process are limited. This study aims to fill this gap by exploring the anti-inflammatory effects of black fig leaf on wound healing and its potential to promote dermal regeneration through histological and genetic analyses. Excisional skin wounds were created in Wistar albino rats, divided into three groups: control (C), cold cream (CC), and 5% black fig leaf cream (FCC). The gene expression levels of cytokines involved in the inflammatory process (interleukin (IL)-1β, IL-6, tumor necrosis factor (TNF)-α) were evaluated using real-time PCR, and protein expression levels were assessed through immunohistochemistry. Histological evaluation of the wound site was performed using hematoxylin and eosin (H&E) and Masson trichrome staining. Molecular analysis showed that the 5% black fig leaf cream exhibited significantly higher anti-inflammatory activity than the control groups. Histopathological examination revealed increased collagen production, angiogenesis, and re-epithelialization, along with reduced inflammatory cell density and bleeding compared to controls. Due to its observed anti-inflammatory activity, the 5% black fig leaf cream may support skin wound healing and has the potential to be a therapeutic agent for skin wounds.

## Introduction

Macrophage polarization is an important pathogenic factor during the inflammatory phase of wound healing. In the early stage of the inflammatory phase, the dominant M1 macrophages lead to the secretion of oxidative metabolites, increased phagocytic activity, and an increase in the expression of pro-inflammatory cytokines such as IL-1α, IL-1β, IL-6, IL-12, and TNF-α to remove pathogens and damaged tissues (Sözmen and Yıldırım Sözmen 2024). Specifically, the increase in pro-inflammatory cytokines prevents infection at the wound site, stimulates cellular recruitment, and activates immune cells (Kanji and Das 2017; Singer and Clark 1999). Furthermore, by activating resident stem and progenitor cells, M1 macrophages promote cell proliferation and differentiation, contributing to the epithelialization phase of wound healing (Larouche et al. 2018).

Approximately 5 days after injury, M2 macrophages replace M1 macrophages, becoming dominant during the tissue repair phase. Anti-inflammatory cytokines such as IL-4, IL-13, and IL-10, which are highly expressed by M2 macrophages, limit inflammation at the wound site, facilitating the transition to the proliferation phase, and promote the production of fibrogenic and angiogenic mediators to support tissue repair (Xu et al. 2020).

Failure to polarize from M1 to M2 macrophages in the wound site leads to the prolonged presence of M1 macrophages and excessive expression of pro-inflammatory cytokines. This imbalance exacerbates extracellular matrix (ECM) disruption, impairs cell migration, and reduces fibroblast proliferation and collagen synthesis. Furthermore, it causes irregularities in the immune response during wound healing, leading to infections caused by various pathogens, increased local necrotic tissue, and poor local vascular conditions (Mast and Schultz 1996). As a result of these imbalances, epithelialization is impaired, leading to difficulties in wound healing and the development of recurrent chronic wounds (Julier et al. 2017; Raziyeva et al. 2021).

Acute and chronic skin wounds, which are a major problem in veterinary medicine as well as in human medicine, pose a significant economic burden on livestock operations. With a better understanding of wound care and the cellular processes involved in wound healing, wound treatment protocols in veterinary medicine have significantly changed over the past 25 years, just as they have in human medicine (Dalgın and Meral 2017). Proper wound care contributes significantly to the normal healing process of wounds. Therefore, wound care should be considered in wound treatment, and studies should be conducted to determine at which stage of wound healing various wound care products might be beneficial (Harding et al. 2002).

Natural bioactive compounds with high levels of antioxidant, anti-inflammatory, and antimicrobial properties can provide significant benefits in the healing of chronic wounds (Schilrreff and Alexiev 2022). It has been shown that different natural plant extracts can be used to develop bioactive wound treatments that provide opportunities to eliminate the inflammatory response and accelerate wound healing (Sharma et al. 2021). As a result of these studies, the molecular and cellular mechanisms involved in the inflammatory phase process can be better understood, potentially enabling the development of more effective treatment protocols and achieving higher healing rates.

The fig tree (*Ficus carica* L.), which grows on all continents and belongs to the Moraceae family, is known for its hepatoprotective, cardioprotective, neuroprotective activities, as well as its antiangiogenic, antihyperlipidemic, antidiabetic, antibacterial, antifungal, antioxidant, and anticarcinogenic effects (Ali et al. 2012; Lansky et al. 2008; Eteraf-Oskouei et al. 2015). Additionally, studies have demonstrated its anti-inflammatory activity (Turkoglu et al. 2017). Dai et al. (2020) showed that *Ficus carica* leaf extract suppressed inflammation caused by denervated muscle atrophy in mice, increased PPARα expression, and alleviated muscle atrophy by inhibiting NF-κB activation. In another study, it was found that the *Ficus carica* leaf extract applied to the submandibular salivary glands of mice injected with 2-nitropropan reduced the gene expression of cytokines IL-6, IL-1β, and TNF-α and regulated inflammation (Ahmed and Abd-El-Hamied 2018). While there are studies demonstrating that fig leaves exhibit anti-inflammatory activity in various tissue injuries and can be used as a therapeutic agent, research on their role in regulating the anti-inflammatory response in skin damage is limited. In this study, the effect of a 5% black fig leaf cream on the release of pro-inflammatory cytokines IL-1β, IL-6, and TNF-α involved in the healing process of skin wounds, as well as its anti-inflammatory role in wound healing, was investigated.

## Materials and methods

### Plant material and extraction

The black fig (*Ficus carica*) leaves used in this study were collected in September 2022 from the Gönen district of Balıkesir province, Türkiye. The collected leaves were washed twice with tap water and once with distilled water, then dried at 25 ± 2°C for 20 days, away from direct sunlight.

The fig leaves were subjected to maceration by adding 70% ethanol and shaking for 48 h. The resulting fig extract was filtered using filter paper, and the filtrate was evaporated under low pressure at 40 °C using a rotary vacuum evaporator (Mopuri et al. 2018). The dark green-colored black fig extract was then mixed with *Unguentum leniens* (which contains 15% wax, 45% liquid paraffin, 1% borax, and distilled water) to prepare a 5% cream for the treatment of clean skin wounds.

### Animal experiments

This study was conducted at the Erciyes University, Hakan Çetinsaya Experimental Research and Application Center (DEKAM), with the approval of Erciyes University Animal Experiments Local Ethics Committee (ERÜ-HADYEK), dated 05/02/2024, decision number 24/016.

In this study, 27 female Wistar albino rats weighing 250–300 g and aged 8 weeks were used. The rats were divided into three groups: control (C, *n* = 9), cold cream (CC, *n* = 9), and 5% black fig leaf cream (FCC, *n* = 9). The rats, housed individually in separate cages, were fed a standard pellet diet and tap water and were maintained under laboratory conditions at 22–24 °C with a 12/12-h light/dark cycle.

Anesthesia was induced intraperitoneally (i.p.) with ketamine hydrochloride (50 mg/kg) and xylazine (10 mg/kg), and circular wounds were created using a 6 mm punch, covering the epidermis. No treatment was applied to the wound areas of the rats in the control group (C) throughout the study, while cold cream was topically applied to the wounds of the rats in the CC group and 5% black fig leaf cream was applied to the wounds of the rats in the FCC group (Pouryousef et al. 2021). The rats in all groups were killed on the 3rd, 7th, and 14th days, and wound tissue samples were collected for histopathological, immunohistochemical, and real-time PCR (qRT-PCR) analyses.

### Histopathologic investigations

Tissue samples obtained from the rats were fixed in 10% formaldehyde solution, then processed through routine procedures and embedded in paraffin blocks. For light microscopic examination, 5 µm-thick sections were obtained from the paraffin blocks and stained with hematoxylin and eosin (H&E) and Masson's trichrome (MT) stains. The stained sections were evaluated using an Olympus BX51 microscope.

### Immunohistochemical staining

An immunohistochemical staining protocol was applied for the determination of three different primary antibodies in the obtained tissues. The primary antibodies used were IL-6 (Bioss, DF6087), TNF-α (Bioss, AF7014), and IL-1β (Bioss, AF5103), with staining performed at the same concentrations for each group. The intensity of immunoreactivity was measured using Image J software (Image J Rasband) at 40× magnification. The following protocol steps were applied for staining.

Initially, the sections were deparaffinized by incubating them for 2 h at 67 °C in an oven, after which they were transferred to xylene at room temperature. The sections were then passed through a decreasing alcohol series (100, 96, 80, 70%) and washed with distilled water. The sections were incubated for 12 min in 3% H_2_O_2_ and then washed with phosphate-buffered saline (PBS). The sections were treated with 5% citrate buffer in a microwave at 600W for 5 min, followed by PBS washing. Afterward, the sections were incubated in 10% goat blocking serum at room temperature for 5 min. The diluted primary antibodies (IL-6, 1:200; TNF-α, 1:100; IL-1β, 1:100; Antibody Diluent Reagent Solution Ref: 003118) were applied to the sections and incubated overnight at + 4 °C. After incubation, the sections were brought to room temperature for 30 min, treated with biotin (Biotinylated goat anti-polyvalent) for 10 min, and washed with PBS. During the streptavidin peroxidase stage, the sections were incubated with a secondary antibody for 10 min. The sections were stained with diaminobenzidine (DAB), followed by Gill’s hematoxylin for 5 min. The sections were then passed through an increasing alcohol series (70, 80, 96, 100%) and incubated in xylene for 30 min. Finally, the slides were mounted with Entellan and visualized under an Olympus BX51 light microscope.

### Gene expression analysis

DNase I (Sigma-Aldrich) treatment was applied to RNA obtained from wound tissues using QIAzol Lysis Reagent to prevent potential DNA contamination. The RNA concentrations (ng/µl) and purity (OD260/280) were determined using a NanoDrop device, and the RNA was calculated to be 1 µg for cDNA synthesis. cDNA synthesis was carried out according to the protocol of the Transcriptor High Fidelity cDNA Synthesis Kit (Roche Ltd., Mannheim, Germany).

qRT-PCR was performed in duplicate using the synthesized cDNA according to the protocol of the FastStart Essential DNA Green Master kit on a LightCycler Nano (Roche Ltd., Mannheim, Germany). The PCR cycling conditions were as follows: 10 min of pre-incubation at 95 °C, followed by 45 amplification cycles (20 s at 95 °C, 20 s at 60 °C, and 20 s at 72 °C). Data for target genes were normalized using housekeeping genes (Table 1). The normalized data were analyzed statistically using the 2^–ΔΔCt^ method (Schmittgen and Livak 2008).

**Table 1:** qRT-PCR expression primers and sequences

Genes	Primer sequences	Reference
IL-1β	F: 5′- CCT CTG ACA GGC AAC CAC TTA -3′ R: 5′- GCA CTG GTC CAA ATT CAA TTC -3′	(Zhang et al. 2019)
IL-6	F: 5′- ATT GTA TGA ACA GCG ATG ATG CAC -3′ R: 5′- CCA GGT AGA AAC GGA ACT CCA GA -3′	(Wang et al. 2020)
TNF-α	F: 5′- CTG GCC ATG GCA TGG ATC TCA AAG A -3′ R: 5′- ATG AAA TGG CAA ATC GGC TGA CGG -3′	(King et al. 2020)
*β*-actin	F: 5′- GCT GTG TTG TCC CTG TAT GC -3′ R: 5′- GAG CGC GTA ACC CTC ATA GA -3′	(Azam et al. 2021)
HPRT1	F: 5′- CTC ATG GAC TGA TTA TGG ACA GGA C -3′ R: 5′- GCA GGT CAG CAA AGA ACT TAT AGC C -3′	(Liu et al., 2021)

### Statistical analysis

Statistical analyses were performed using SPSS 22 data analysis software. The normality of data distribution was assessed using the Shapiro–Wilk test. One-way analysis of variance (ANOVA) was applied to compare groups for variables showing normal distribution, and if a significant difference was found, post hoc multiple group comparisons were performed using the Tukey test. Data are presented as mean ± standard error. A *P* value of < 0.05 was considered statistically significant.

## Results

### Histopathological study findings

**Fig. 1:**
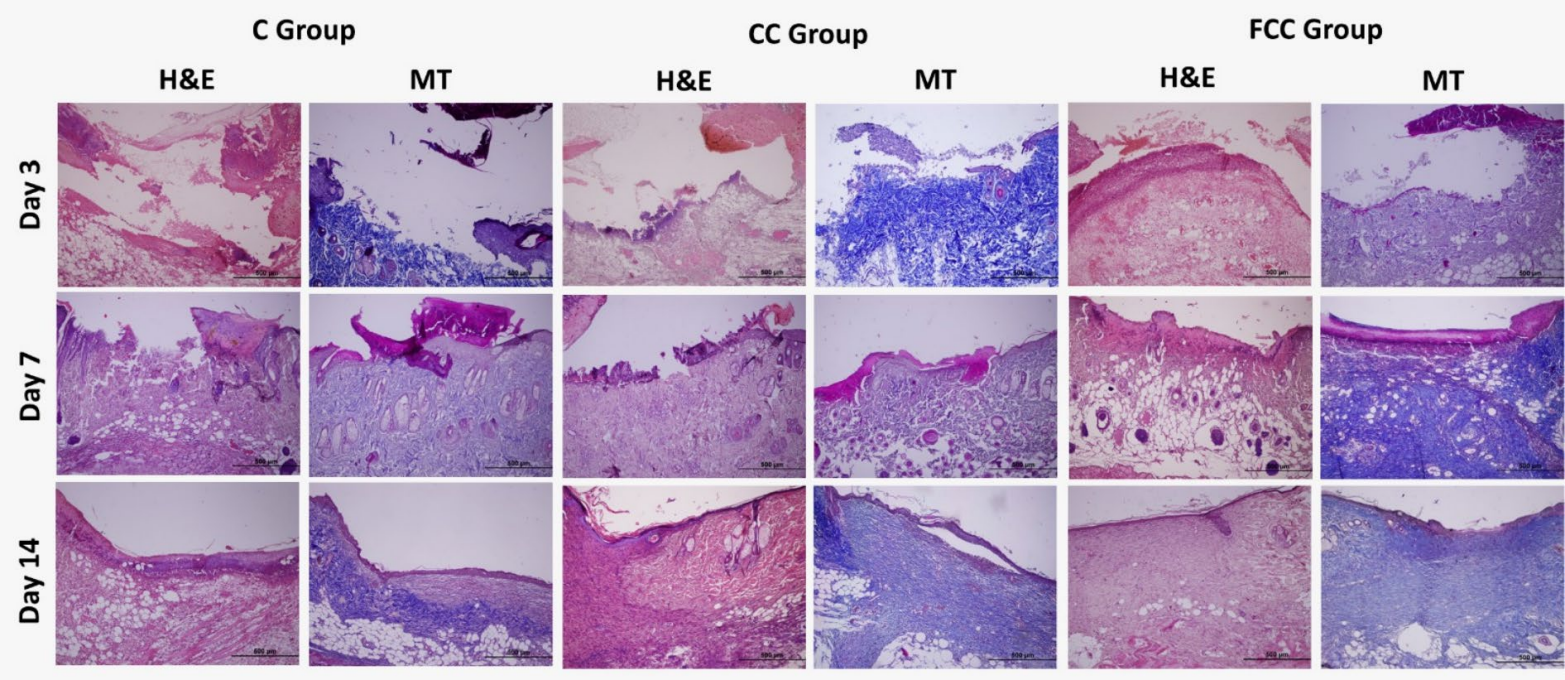
H&E and MT staining images of the C, CC, and FCC groups on days 3, 7, and 14 (×10)

The general histological structures of the wound tissues from the C, CC, and FCC groups on days 3, 7, and 14 were examined using H&E and MT staining (Fig. 1).

Histopathological evaluation of the wound region on day 3 revealed that, compared to the C and CC groups, the FCC group exhibited less inflammatory cell infiltration and hemorrhagic areas within the connective tissue. In the FCC group, re-epithelialization was notably prominent at the edges of the wound lips, with a higher density of coarse/dense (Type III) collagen formation. Additionally, areas of neovascularization, either linear or round in shape, were observed.

Histopathological evaluation of the wound region on day 7 revealed that in the FCC group, compared to day 3, there was a significant reduction in the presence of mononuclear cell infiltration and hemorrhagic areas, dense collagen fibers exhibited an areolar appearance, and the formation of new blood vessels increased. In contrast, in the C and CC groups on day 7, beneath the wound crust, inflammatory foci predominantly composed of neutrophils and necrotic areas were present. The inward concave slope resulting from the punch wound persisted, and although collagen fiber formation was not yet markedly evident in the wound area, re-epithelialization, which was not observed on day 3, began at the wound lips. Additionally, an increase in fibroblastic activity was noted, along with the slight formation of new blood vessels.

Histopathological evaluation of the wound region on day 14 revealed that in the C and CC groups, the wound areas had contracted, with the formation of an immature epithelial layer in the majority of cases. The wound lips had elongated and moved closer to one another, with dense collagen fibers beginning to show an areolar appearance. Mononuclear cell infiltration, angiogenesis, and hemorrhagic areas remained present between these fibers. In the FCC group on day 14, however, the wound areas were completely closed, with the formation of two to three layers of thin epithelial cells in both mature and immature epithelial layers. Papillae began to form beneath the epithelial tissue, and a thin keratin layer appeared on the surface of the epithelial layer. Moreover, in the FCC group, collagen fibers nearly completely covered the wound area. The mononuclear cell infiltration, inflammatory cell infiltration, and angiogenesis observed on day 7 between the collagen fibers were no longer evident, and hair follicles were observed to begin forming in some areas.

### Immunohistochemical study findings

Immunohistochemical staining was used to determine the activities of the inflammatory cytokines IL-1β, IL-6, and TNF-α, which are important markers of inflammation, in the wound area. In the staining performed with the DAB chromogen, immunopositive areas for IL-1β, IL-6, and TNF-α were prominently highlighted in brown. The intensity and distribution of the staining were quantified using the Image J software program. According to the analysis results, it was observed that the protein expressions of IL-1β, IL-6, and TNF-α (Table 2) were particularly prominent in areas close to the wound region in the C, CC, and FCC groups.

**Table 2:** Protein expression levels of IL-1β, IL-6, and TNF-α in the study groups and statistical values

		C	CC	FCC	*p*
IL1-β	Day 3	92.25 ± 8.09^a^	79.35 ± 3.70^b^	60.20 ± 4.06^c^	0.001***
	Day 7	61.60 ± 4.01^a^	37.75 ± 5.35^b^	27.00 ± 5.20^c^	0.001***
	Day 14	75.30 ± 3.06^a^	56.85 ± 6.58^b^	32.20 ± 4.90^c^	0.001***
IL-6	Day 3	104.85 ± 7.09^a^	101.10 ± 5.42^a^	100.20 ± 7.36^a^	0.074
	Day 7	96.10 ± 5.19^a^	94.05 ± 5.92^a^	67.00 ± 5.61^b^	0.001***
	Day 14	93.40 ± 6.35^a^	78.65 ± 7.02^b^	37.95 ± 5.17^c^	0.001***
TNF-α	Day 3	103.20 ± 6.84^a^	87.50 ± 4.82^b^	75.35 ± 4.85^c^	0.001***
	Day 7	100.30 ± 6.17^a^	74.75 ± 6.06^b^	57.45 ± 8.46^c^	0.001***
	Day 14	98.65 ± 8.68^a^	62.75 ± 7.41^b^	32.40 ± 5.04^c^	0.001***

Statistical significance is considered for values of *p < 0.05, **p < 0.01, and ***p < *0.001. Identical letters in the same row indicate similarity between the groups, while different letters represent a significant difference between the groups*

**Fig. 2:**
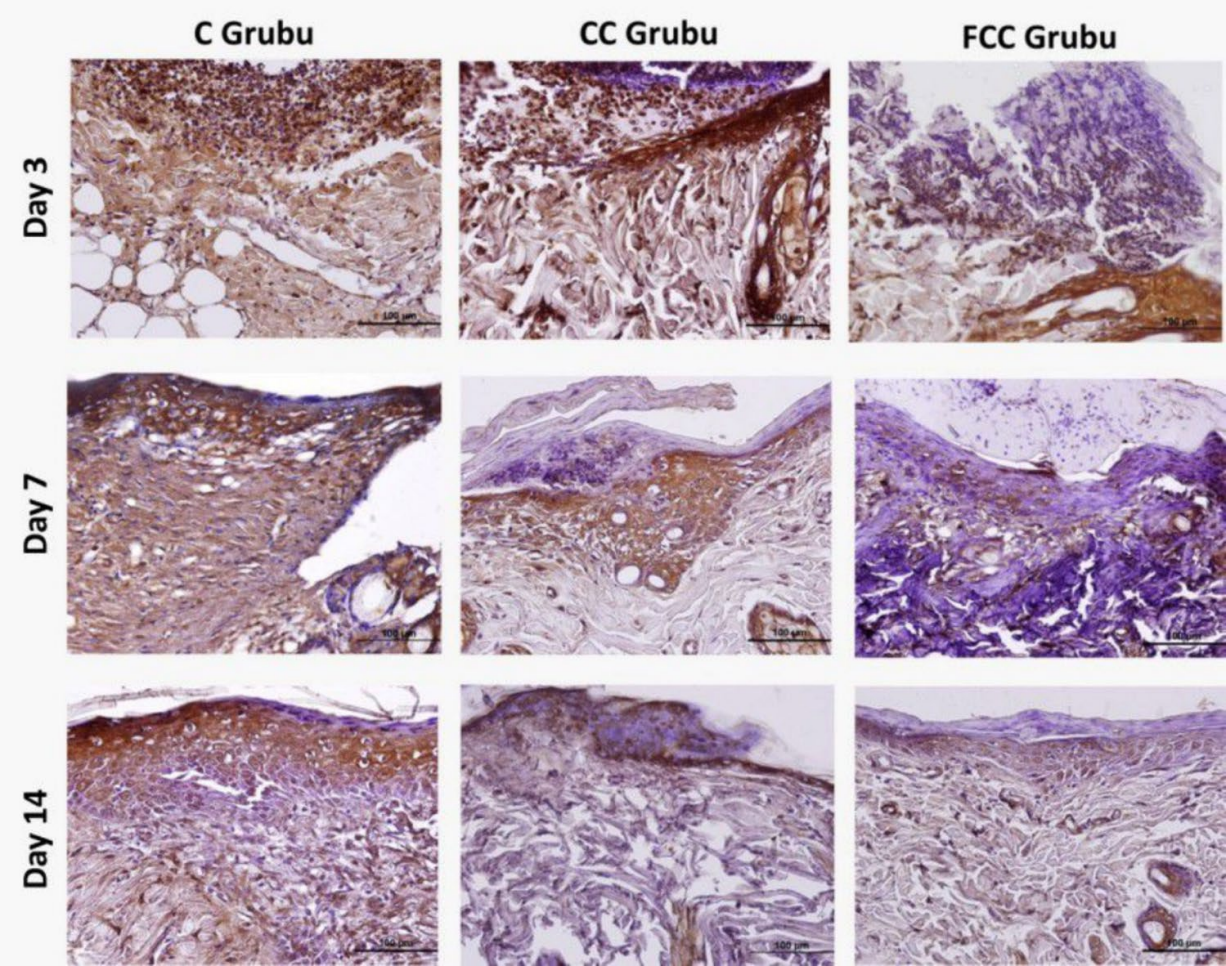
IL-1β protein expression in the study groups (areas with IL-1β immunoreactivity show a brown reaction, ×40)

Following wound formation, on day 3, it was observed that IL-1β protein expression was increased in the C group compared to the CC and FCC groups, and this increase in expression levels was statistically significant (*p* < 0.05). On day 3, the FCC group showed a statistically significant decrease in IL-1β protein expression intensity compared to the other groups (*p* < 0.05). In both day 7 and day 14 tissue sections, the highest IL-1β protein expression intensity was observed in the C group, with a decrease in IL-1β expression in the CC and FCC groups. Statistical analysis of pairwise comparisons between the groups revealed that this decrease was statistically significant (*p* < 0.05). In conclusion, the highest IL-1β protein expression intensity was found in the C group, the lowest in the FCC group, and expression levels gradually decreased in all groups from day 3 to day 14 (Fig. 2).

**Fig. 3:**
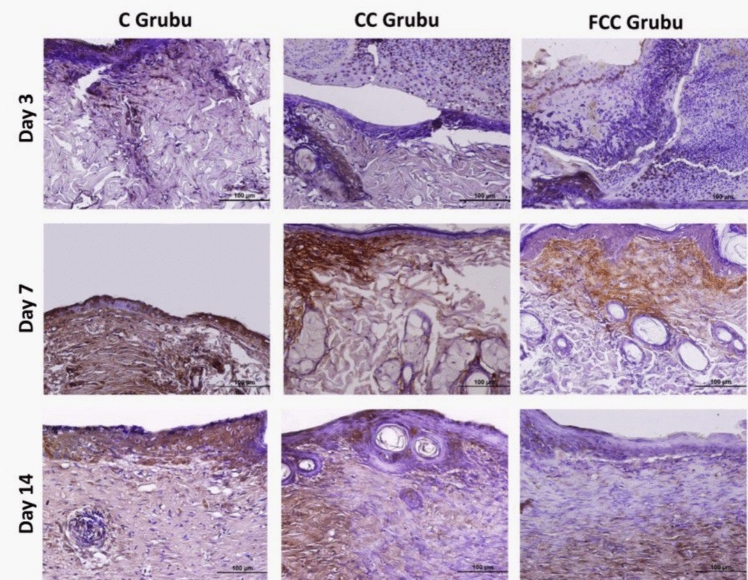
IL-6 protein expression in the study groups (areas with IL-6 immunoreactivity show a brown reaction, ×40)

Following wound formation, on day 3, IL-6 protein expression was high in all groups; however, no statistically significant differences were observed between the groups (*p* > 0.05). In contrast, on day 7, IL-6 protein expression intensity was similar in the C and CC groups, while the IL-6 expression intensity in the FCC group was significantly lower compared to the other two groups, and this decrease was statistically significant (*p* < 0.05). On day 14, IL-6 protein expression intensity was found to be statistically significantly lower in the FCC group compared to the C and CC groups (*p* < 0.05). In conclusion, the highest IL-6 expression was observed in the C group, the lowest in the FCC group, and IL-6 expression gradually decreased across all groups from day 3 to day 14 (Fig. 3).

**Fig. 4:**
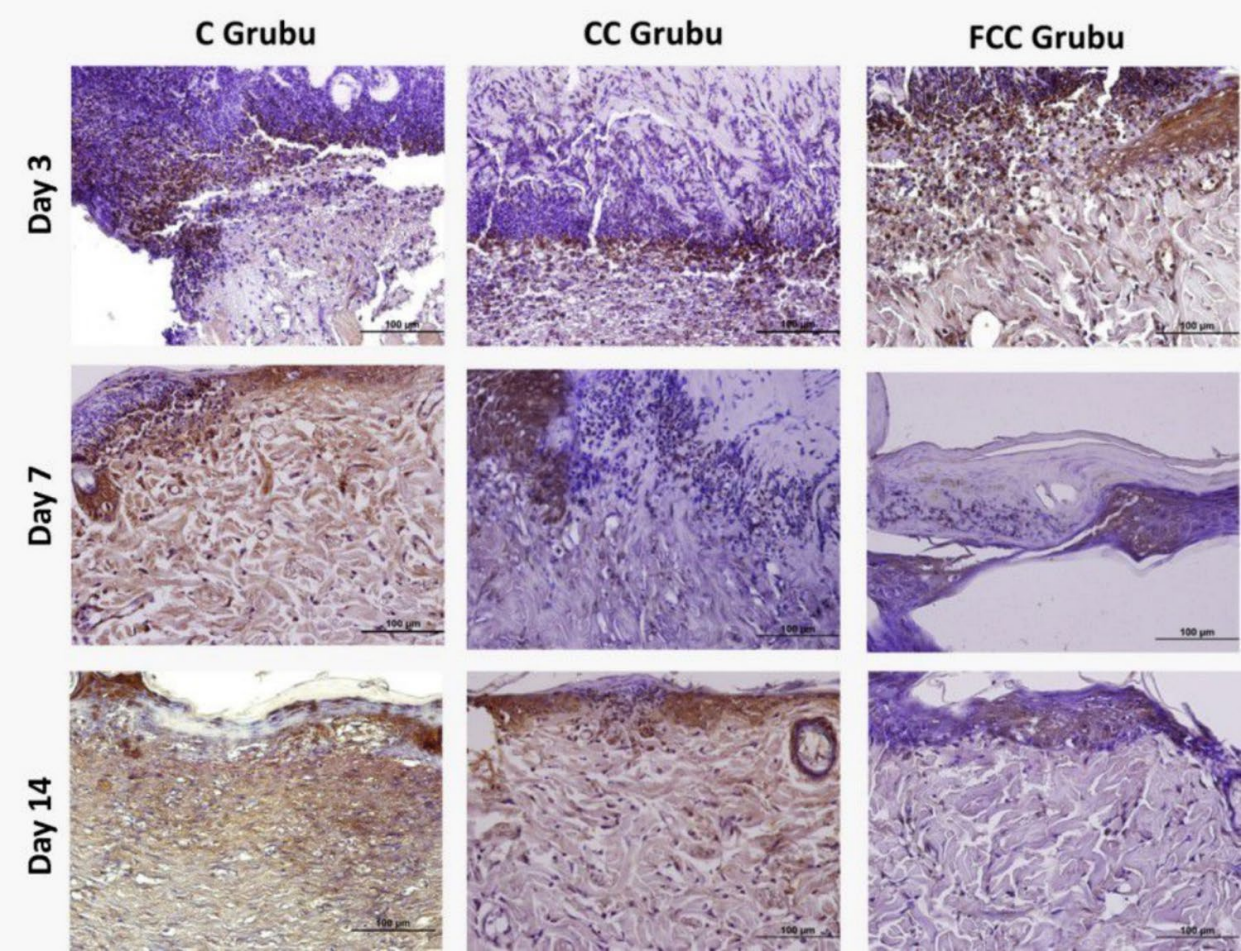
TNF-α protein expression in the study groups (areas with TNF-α immunoreactivity show a brown reaction, ×40)

Following wound formation, on day 3, TNF-α protein expression showed a significant increase in the C group compared to the CC and FCC groups, with the expression intensity of TNF-α in the FCC group being lower than in the other two groups. These results were statistically significant (*p* < 0.05). In contrast, on days 7 and 14, the highest expression level was observed in the C group, while TNF-α expression was lower in the CC and FCC groups. Statistical analysis of pairwise comparisons revealed a significant difference between the groups (*p* < 0.05). In conclusion, the highest TNF-α expression level was found in the C group, the lowest in the FCC group, and expression levels decreased gradually in all groups from day 3 to day 14 (Fig. 4).

### Gene expression findings

The gene expression levels of IL-1β, IL-6, and TNF-α were normalized with the housekeeping genes *β*-actin and HPRT1, followed by comparison with the control groups to determine the increase or decrease in gene expression levels across all groups.

**Fig. 5:**
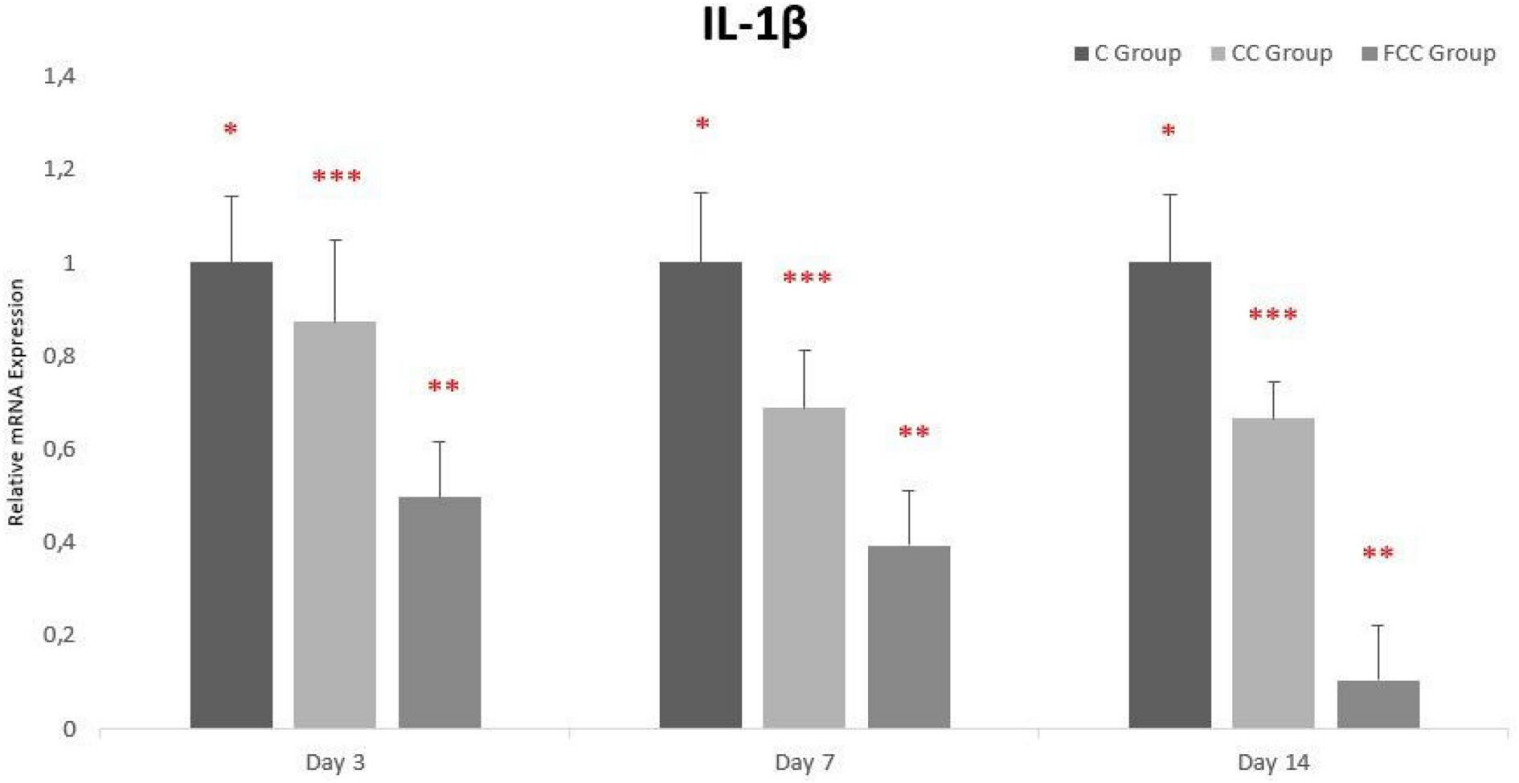
Gene expression analysis of IL-1β in the study groups. *p < 0.05, **p < 0.01, and ***p < 0.001 were considered statistically significant

When evaluating IL-1β gene expression levels between days and groups, it was determined that the highest IL-1β expression levels were observed in the C group and the lowest levels in the FCC group. From day 3 to day 14, IL-1β gene expression gradually decreased in all groups, and this decrease was statistically significant across all groups (Fig. 5).

**Fig. 6:**
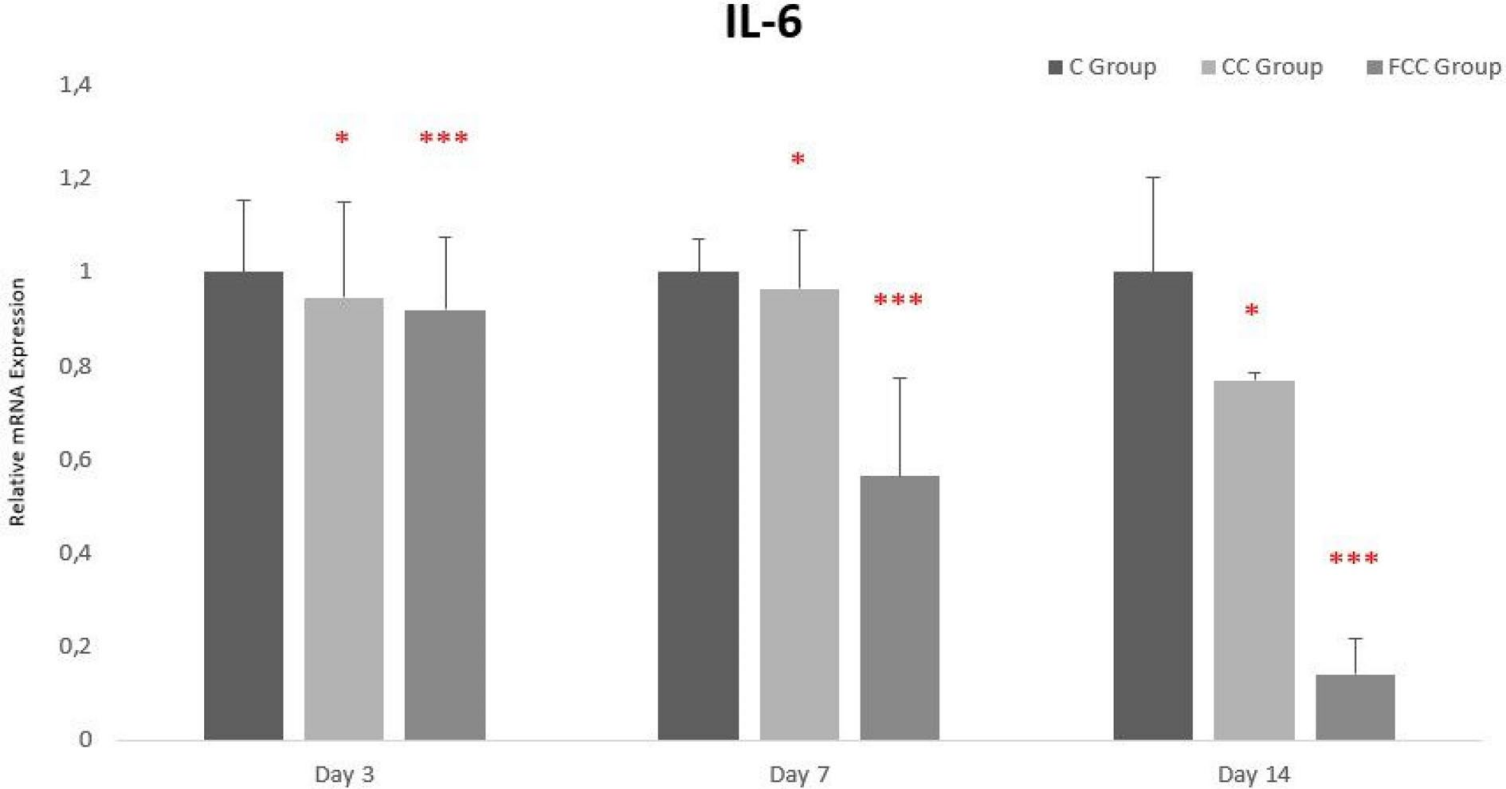
Gene expression analysis of IL-6 in the study groups. *p < 0.05, **p < 0.01, and ***p < 0.001 were considered statistically significant

When evaluating IL-6 gene expression levels between days and groups, expression levels were similar across all groups on day 3. On day 7, IL-6 gene expression levels were similar in the C and CC groups, but expression levels in the FCC group had decreased. On day 14, the highest IL-6 expression level was observed in the C group and the lowest in the FCC group. Overall, from day 3 to day 14, IL-6 gene expression gradually decreased across all groups, with this decrease being statistically significant in the CC and FCC groups (Fig. 6).

**Fig. 7:**
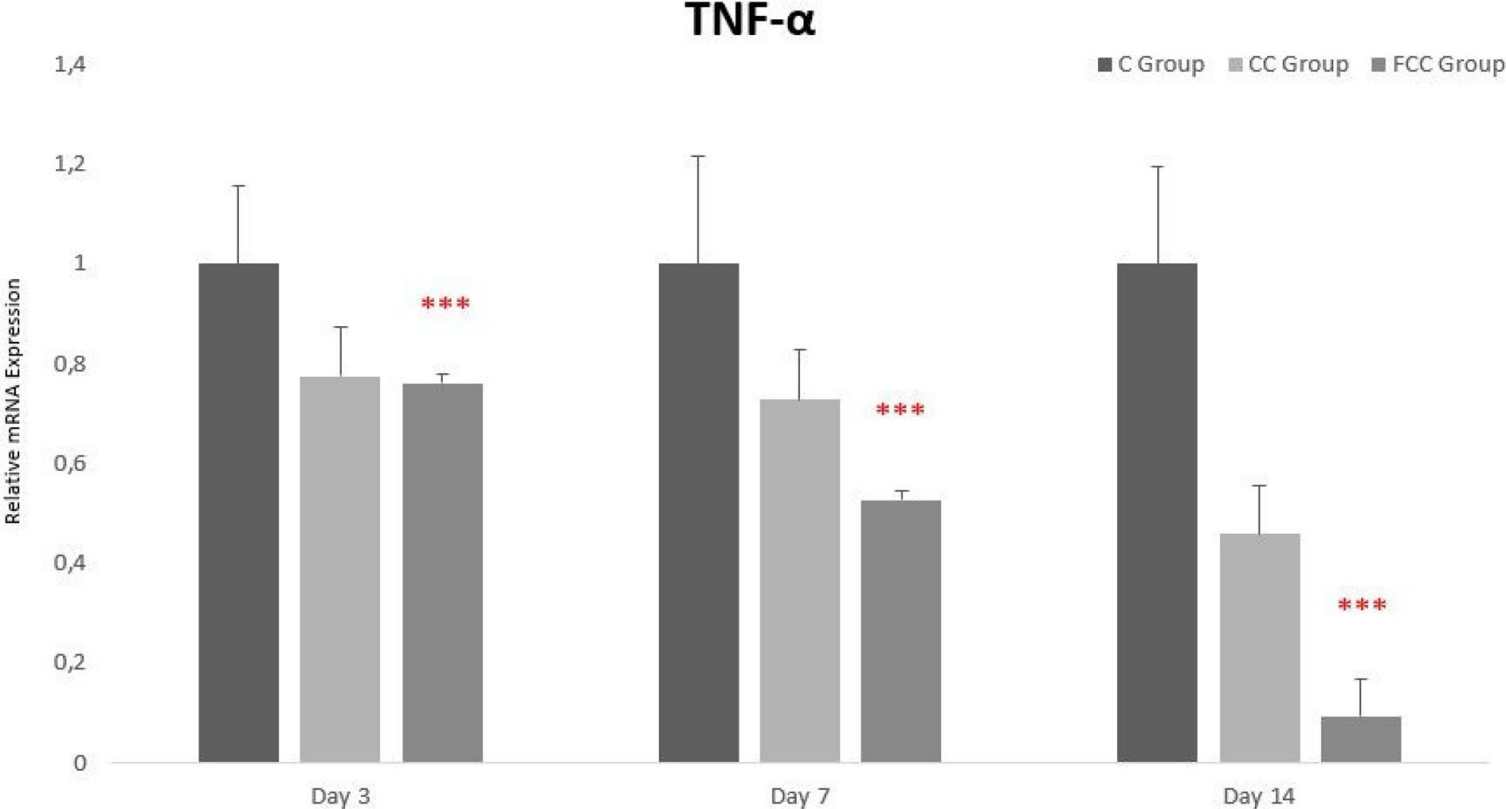
Gene expression analysis of TNF-α in the study groups. *p < 0.05, **p < 0.01, and ***p < 0.001 were considered statistically significant

When evaluating TNF-α gene expression levels between days and groups, the highest TNF-α expression levels were observed in the C group, and the lowest in the FCC group on days 3, 7, and 14. Overall, from day 3 to day 14, TNF-α gene expression gradually decreased across all groups, with this decrease being statistically significant only in the FCC group (Fig. 7).

## Discussion

Inflammation is the natural defense response of the immune system to pathogens or endogenous molecules that are not pathogenic. During this process, inflammation is regulated by pro-inflammatory cytokines such as TNF-α, IL-1β, and IL-6, as well as mediators like nitric oxide (NO) and prostaglandin E2 (PGE2) (Yi et al. 2017).

Dysregulation of cytokines involved in the inflammatory process leads to chronic inflammatory diseases that require long-term treatment. Diseases associated with chronic inflammation pose a significant threat to both human and animal health, which makes the development of effective therapeutic strategies to regulate inflammatory responses critically important (Yi et al. 2017; Ginwala et al. 2019). In this context, numerous studies have investigated the effects of various plant extracts on the inflammatory response during the wound healing process. In a study conducted by Fan et al. (2019), extracts obtained from *Coptis chinensis* rhizomes, *Scutellaria baicalensis* roots, *Phellodendron amurense* bark, and *Gardenia jasminoides* fruits were applied to mouse skin tissues with induced atopic dermatitis-like lesions and LPS-stimulated RAW264.7 cells. In the extract-treated mice, a significant reduction in atopic dermatitis-like lesion symptoms, alleviation of inflammatory mediator infiltration, a decrease in IL-1α serum expression levels, normalization of IL-1β, IL-6, and TNF-α serum levels, and inactivation of the MAPK (including p38, ERK, and JNK), IκB-α, and NF-κB (p65) pathways were observed. Additionally, a marked decrease in the protein and mRNA expression levels of IL-1α, IL-1β, IL-6, and TNF-α was also noted in RAW264.7 cells. In another study, evening primrose oil (EPO) emulsion, rich in omega-3, omega-6, and omega-9 fatty acids, was topically applied to excisional wounds created in rats. In the treatment groups, compared to the control groups, increased expression of VEGF and type I collagen, accelerated wound closure, and significant improvement on day 14 were observed. Additionally, in the early stages of healing, a gradual decrease in the expression of pro-inflammatory cytokines IL-1β, IL-6, and TNF-α was noted from day 3 to day 14 (Ishak et al. 2018). In another study, a cream containing *Acacia nilotica* extract significantly increased wound contraction rate, capillary formation, and re-epithelialization on days 7 and 14 in rats while also gradually suppressing IL-1β and TNF-α expression (Kankara et al. 2017). Similarly, *Sophora alopecuroides* extract gel gradually reduced IL-1β and TNF-α expression in rat skin wounds on days 7 and 12 (Sun et al. 2021). A 10% *Bergenia ciliata* ointment was observed to increase collagen formation, re-epithelialization, and neovascularization in rats while decreasing IL-6 and TNF-α expression levels (Kour et al. 2021). In another study, excisional skin wounds involving the epidermis, dermis, and hypodermis in mice were treated with water as a control and olive oil for treatment purposes. In the treatment groups, inflammatory cell infiltration and TNF-α gene and protein expression levels in the wound region decreased from day 3 to day 7, while re-epithelialization, blood vessel count, collagen accumulation, myofibroblast differentiation, and wound contraction were higher in the olive oil group compared to the control (Donato-Trancoso et al. 2016).

Compared to studies investigating inflammation in the wound healing process using herbal products, research on the effects of fig leaf, known for its anti-inflammatory properties, on the inflammation process in dermal regeneration is quite limited. In a study conducted by Gültekin Tosun (2024), it was reported that the application of 5% black fig leaf cream accelerated wound healing in rat skin wound areas using a punch biopsy model by increasing the gene and protein expression levels of type 1 collagen, responsible for collagen formation, and VEGF, involved in angiogenesis and re-epithelialization (Gültekin Tosun 2024). In this study, however, it was found that the application of 5% black fig leaf cream reduced the gene and protein expression levels of pro-inflammatory cytokines, IL-1β, IL-6, and TNF-α, suppressed inflammation, and simultaneously accelerated re-epithelialization, collagen deposition, and vessel formation in the skin wound area. As a result, it was determined that faster wound healing was achieved in the wound area due to the anti-inflammatory effect of 5% black fig leaf extract.

In line with the findings of this study, a study conducted by Begum et al. (2013) reported that in rats with an excisional wound model, the application of 10% fig leaf ointment resulted in the disappearance of edema and inflammatory exudates in the skin wound compared to the control groups. It also promoted early dermal and epidermal regeneration, re-epithelialization, collagen formation, and the development of new blood vessels. Furthermore, a study by Lee ve Lee (2023) demonstrated that *Ficus carica* fruit extract (FFE) exhibited anti-inflammatory activity by reducing NO production and iNOS expression in macrophage (RAW264.7) cells in LPS-induced BALB/c mice. This treatment alleviated symptoms such as dryness, scaling, and thickened epidermis in skin lesions caused by psoriasis.

A good wound healing process is only possible with a decrease in pro-inflammatory cytokines, thus regulating inflammation. In this study, it was observed that the 5% black fig leaf extract reduced the gene and protein expression of pro-inflammatory cytokines such as IL-1β, IL-6, and TNF-α following skin injury. In a previous study by Rezagholizadeh et al. (2022), it was reported that the phenolic compounds, steroidal saponins, ficusogenin, bergapten, lupeol, and psoralen, which are found in fig leaves, inhibit pro-inflammatory cytokines and exhibit anti-inflammatory effects. Based on these studies, it is believed that the rich phytochemicals in the 5% black fig leaf extract in our research reduce the gene and protein expression of IL-1β, IL-6, and TNF-α. As a result, the anti-inflammatory potential of the 5% black fig leaf extract demonstrates its potential to accelerate the healing process in skin wounds.

## Conclusion

In this study, the effect of 5% black fig leaf extract on the gene and protein expression of IL-1β, IL-6, and TNF-α cytokines involved in the healing of skin wounds was examined for the first time. Histopathological analyses and gene and protein expression analyses revealed that the black fig leaf extract has an anti-inflammatory effect. Both this study and the research by Gültekin Tosun (2024) suggest that black fig leaf extract can be used as a natural product in the treatment of skin wounds. However, to further elucidate the anti-inflammatory activity of black fig leaf, it is believed that the effects of genes involved in the regulation of pro-inflammatory cytokines, such as NF-κB, COX-2, PGE2, and signaling pathways like mitogen-activated protein kinases (MAPK), extracellular signal-regulated kinases (ERK1/2), and Janus kinases/signal transducers and activators of transcription (JAK/STAT) should also be investigated. Furthermore, comprehensive research will be crucial to identify which phytobiological compounds in black fig leaves are most prominent in exerting anti-inflammatory effects.

## Data Availability

Data will be made available on request.

## Abbreviations

ACTB: Beta-actin
DAB: Diaminobenzidine
ECM: Extracellular matrix
HPRT1: Hypoxanthine phosphoribosyltransferase 1
IL-1β: Interleukin 1 beta
IL-6: Interleukin 6
MAPK: Mitogen-activated protein kinase
NF-κB: Nuclear factor kappa B
NO: Nitric oxide
PGE2: Prostaglandin E2
TNFα: Tumor necrosis factor alpha
VEGF: Vascular endothelial growth factor
